# Attenuation of corneal myofibroblast development through nanoparticle-mediated soluble transforming growth factor-β type II receptor (sTGFβRII) gene transfer

**Published:** 2012-10-20

**Authors:** Ajay Sharma, Jason T. Rodier, Ashish Tandon, Alexander M. Klibanov, Rajiv R. Mohan

**Affiliations:** 1 Harry S. Truman Memorial Veterans' Hospital, Columbia, MO; 2 Mason Eye Institute, School of Medicine, University of Missouri, Columbia, MO; 3 Department of Chemistry, Massachusetts Institute of Technology, Cambridge, MA; 4 Department of Biological Engineering, Massachusetts Institute of Technology, Cambridge, M; 5 College of Veterinary Medicine, University of Missouri, Columbia, MO

## Abstract

**Purpose:**

To explore (i) the potential of polyethylenimine (PEI)-DNA nanoparticles as a vector for delivering genes into human corneal fibroblasts, and (ii) whether the nanoparticle-mediated soluble extracellular domain of the transforming growth factor–β type II receptor (sTGFβRII) gene therapy could be used to reduce myofibroblasts and fibrosis in the cornea using an in vitro model.

**Methods:**

PEI-DNA nanoparticles were prepared at a nitrogen-to-phosphate ratio of 30 by mixing linear PEI and a plasmid encoding sTGFβRII conjugated to the fragment crystallizable (Fc) portion of human immunoglobulin. The PEI-DNA polyplex formation was confirmed through gel retardation assay. Human corneal fibroblasts (HCFs) were generated from donor corneas; myofibroblasts and fibrosis were induced with TGFβ1 (1 ng/ml) stimulation employing serum-free conditions. The sTGFβRII conjugated to the Fc portion of human immunoglobulin gene was introduced into HCF using either PEI-DNA nanoparticles or Lipofectamine. Suitable negative and positive controls to compare selected nanoparticle and therapeutic gene efficiency were included. Delivered gene copies and mRNA (mRNA) expression were quantified with real-time quantitative PCR (qPCR) and protein with enzyme-linked immunosorbent assay (ELISA). The changes in fibrosis parameters were quantified by measuring fibrosis marker α-smooth muscle actin (SMA) mRNA and protein levels with qPCR, immunostaining, and immunoblotting. Cytotoxicity was determined using cellular viability, proliferation, and terminal deoxynucleotidyl transferase dUTP nick end labeling (TUNEL) assay.

**Results:**

PEI readily bound to plasmids to form nanoparticular polyplexes and exhibited much greater transfection efficiency (p<0.01) than the commercial reagent Lipofectamine. The PEI-DNA-treated cultures showed 4.5×10^4^ plasmid copies/µg DNA in real-time qPCR and 7,030±87 pg/ml sTGFβRII protein in ELISA analyses, whereas Lipofectamine-transfected cultures demonstrated 1.9×10^3^ gene copies/µg DNA and 1,640±100 pg/ml sTGFβRII protein during these assays. The PEI-mediated sTGFβRII delivery remarkably attenuated TGFβ1-induced transdifferentiation of corneal fibroblasts to myofibroblasts in cultures, as indicated by threefold lower levels of SMA mRNA (p<0.01) and significant inhibition of SMA protein (up to 96±3%; p<0.001 compared to no-gene-delivered cultures) in immunocytochemical staining and immunoblotting. The nanoparticle-mediated delivery of sTGFβRII showed significantly better antifibrotic effects than the Lipofectamine under similar experimental conditions. However, the inhibition of myofibroblast in HCF cultures by sTGFβRII overexpression by either method was significantly higher than the naked vector transfection. Furthermore, PEI- or Lipofectamine-mediated sTGFβRII delivery into HCF did not alter cellular proliferation or phenotype at 12 and 24 h post-treatment. Nanoparticles treated with HCF showed more than 90% cellular viability and very low cell death (2–6 TUNEL+ cells), suggesting that the tested doses of PEI-nanoparticles do not induce significant cell death.

**Conclusions:**

This study demonstrated that PEI-DNA nanoparticles are an attractive vector for the development of nonviral corneal gene therapy approaches and that the sTGFβRII gene delivery into keratocytes could be used to control corneal fibrosis in vivo.

## Introduction

Nanomedicine is an emerging field for developing long-term sustained and effective therapies for various diseases. Due to their diminutive size and unique physical and chemical properties, nanoparticles can readily enter the target cells and deliver therapeutic payloads in the form of DNA, proteins, or drugs. Many recent studies have reported the gene transfer ability of numerous metallic and nonmetallic nanoparticles in a variety of cells [1-4]. The potential of nanoparticles such as gold, albumin, 1,2-dioleoyl-3-trimethylammonium-propane, 1,2-dioleoyl-sn-glycero-3-phosphoethanolamine, and poly(lactic-co-glycolic acid) for developing nonviral corneal gene therapy approaches has been recently reported [5-7]. Polyethylenimine (PEI) is a polycation that has shown high gene transfer efficiency in many cell types [8-11]; yet its potential for corneal gene therapy has not been tested. PEI efficiently condenses DNA to form stable functionalized nanoparticles [12]. After their cellular uptake, PEI’s proton sponge effect facilitates gene expression by causing efficient DNA release from endosomes due to proton and chloride influx, thus leading to endosome rupture by osmotic swelling [13]. Branched and linear PEIs are available; although both show efficient gene transfer, linear PEI is less toxic in vivo [14,15]. In particular, a 22 kDa linear PEI has been shown to have high transfection efficiency both in vitro and in vivo [11-15].

Corneal fibrosis is an expected outcome of uncontrolled wound healing following injury or infection. Corneal healing is an intricate process involving increased cytokine expression, keratocyte activation, myofibroblast formation, and increased extracellular matrix (ECM) deposition [16-19]. Uneven ECM deposition and formation of light-scattering myofibroblasts are thought to be the underlying causes of corneal fibrosis [18,19], with TGFβ as a key cytokine involved in the pathophysiology of corneal fibrosis due to its increased expression in tear fluid and stroma in injured corneas [20,21]. In vitro TGFβ treatment of corneal keratocytes or fibroblasts induces myofibroblast formation, stimulates cytoskeletal and ECM protein synthesis, and reduces ECM degradation by decreasing matrix metalloproteinases, collagenase, and stromelysin expression [22-24]. Silencing of hyper-TGFβ activity with small molecule inhibitors, genes, antisense oligos, or neutralizing antibodies is effective in preventing corneal fibrosis in various animal models [25-29].

It has been our longstanding central hypothesis that selective sequestering of TGFβ by anti-TGFβ genes is an attractive approach to treat corneal fibrosis. We found that anti-TGFβ gene therapy delivered with nonpathogenic adeno-associated viruses effectively reduced corneal fibrosis in vivo in a rabbit model [29], but little research has been done to identify potent nonviral vehicles such as nanoparticles for corneal disease treatment. Gene-based therapy eliminates many challenges of conventional therapy, including repeated applications and side effects. In the present study, we report PEI-DNA nanoparticles as a vector for introducing therapeutic genes into corneal cells and the effectiveness of soluble extracellular domain of the TGFβ type II receptor to inhibit TGFβ-induced formation of myofibroblasts in the cornea using an in vitro model.

## Methods

### Human corneal fibroblast and myofibroblast cultures

Primary corneal fibroblast cultures were generated from donor human corneas procured from an eye bank (Heartland Eye Bank, Kansas City, MO) as reported previously [28]. Briefly, corneal tissues were washed with sterile cell culture medium, and the epithelium and endothelium were removed by gentle scraping with a scalpel blade (#15). The corneal stroma was cut into small pieces, placed on culture dish and incubated in a humidified 5% CO_2_ incubator at 37 °C in Dulbecco’s modified Eagle’s medium (DMEM) supplemented with 10% fetal bovine serum for 2 weeks or longer. The primary human corneal fibroblasts (HCFs) harvested from these corneal buttons were seeded in six-well plates in DMEM supplemented with 10% fetal bovine serum and allowed to reach 60%–70% confluence. To generate myofibroblast cultures, HCFs were seeded using DMEM containing 10% serum, after 8–12 h switched to serum-free medium containing TGFβ1 (1 ng/ml), and incubated for 4–7 days. The cultures were fed with fresh serum-free TGFβ1 containing medium every 24 h.

### Vector construct, polyethylenimine-DNA nanoparticles, and transfection

The 477-nucleotide sequence encoding for extracellular domain of human TGFβRII (sTGFβRII) conjugated to a 681 intronless nucleotide sequence encoding for a fragment crystallizable (Fc) portion of human immunoglobulin (IgG) was cloned into pcDNA3.1 mammalian gene expression vector, as reported previously [30]. Restriction mapping and DNA sequencing were used to confirm the nucleotide sequence of the construct.

The PEI-DNA nanoparticle transfection mixture (nitrogen-to-phosphate ratio 30) was prepared by adding appropriate amounts of 150 mM linear 22 kDa PEI [31] in 100 μl of water dropwise with constant stirring to 2 μg of plasmid (pcDNA3.1-sTGFβRII-Fc) solubilized in 100 μl of PBS containing 10% glucose (w/v). The reaction mixture was then incubated at 37 °C for 30 min. The PEI plasmid DNA complexation was confirmed using a gel retardation assay by loading onto a 1% agarose gel containing ethidium bromide and subjecting to electrophoresis with a Tris-acetate running buffer. The Lipofectamine transfection solution was prepared by incubating 5 µl Lipofectamine 2000 (Invitrogen, Carlsbad, CA) in 250 µl of serum-free DMEM with 2 µg of DNA in 250 µl of serum-free DMEM for 30 min at room temperature.

Transfections of HCF were performed by incubating HCF cultures with 200 μl of PEI-DNA solution in 2 ml DMEM medium containing 10% serum for 30 min or Lipofectamine-DNA mixture in 2 ml DMEM serum-free medium for 6 h (as recommended by the vendor), since no DNA delivery was detected after 30 min incubation. After transfection-solution incubation, cultures were washed with PBS and allowed to grow in serum-free DMEM medium containing TGFβ1 (1 ng/ml) for 4–7 days (80% confluence).

### Immunofluorescence

Cells were fixed with 4% paraformaldehyde, and immunofluorescence staining for α–smooth muscle actin (αSMA; a marker for myofibroblast) was performed. Samples were incubated with 5% BSA for 30 min at room temperature, followed by mouse monoclonal αSMA antibody (1:200 dilution, Dako, Carpinteria, CA) for 90 min and Alexa 488 goat anti-mouse IgG secondary antibody (1:500 dilution) for 1 h. The cells were washed three times in HEPES buffer, mounted in Vectashield containing 4'-6-diamidino-2-phenylindole (DAPI; Vector Laboratories), and viewed and photographed with a Leica fluorescent microscope (Leica DM 4000B) equipped with a digital camera (SpotCam RT KE).

### Immunoblotting

The treated/untreated HCF cultures were lysed in radioimmunoprecipitation assay (RIPA) lysis buffer containing a protease inhibitor cocktail (Roche Applied Sciences, Indianapolis, IN), followed by centrifugation at 10,000 g for 10 min. Samples were suspended in NuPAGE LDS buffer containing a reducing agent (Invitrogen) and heated at 70 °C for 10 min. Protein samples were resolved by NuPAGE Novex Bis-Tris mini gels (Invitrogen) and transferred onto the polyvinylidene difluoride membranes using an iBlot apparatus (Invitrogen). The transferred proteins were detected by incubating the membrane with primary antibodies: αSMA (Dako) and glyceraldehyde-3-phosphate dehydrogenase (GAPDH; Santa Cruz Biotechnology, Santa Cruz, CA), followed by alkaline phosphatase conjugated anti-mouse secondary antibody. After washing three times in 0.05% Tween-20 in Tris-buffered saline of pH 8.0 for 5 min each, the blot was developed using the nitroblue tetrazolium/5-bromo-4-chloro-3-indolylphosphate method. Three separate western blots were performed for each experiment. The digital quantification of western blots was performed using Image J software. The quantification intensity was normalized against GAPDH bands.

### RNA extraction, cDNA synthesis, and quantitative real-time PCR

Total RNA from cells was extracted with an RNeasy kit (Qiagen, Valencia, CA) and reverse transcribed to cDNA following vendor’s instructions (Promega, Madison, WI). Real-time PCR was then performed using Step One Plus real-time PCR system (Applied Biosystems, Carlsbad, CA). A 20-µl reaction mixture containing 2 µl of cDNA, 2 µl of forward primer (200 nM), 2 µl of reverse primer (200 nM), and 10 µl of 2X SYBR green super mix (Bio-Rad Laboratories) was run at a universal cycle (95 °C for 10 min, 40 cycles at 95 °C for 15 s, and 60 °C for 60 s) in accordance with the manufacturer’s instructions. For alpha smooth muscle actin (αSMA), the forward primer sequence TGG GTG ACG AAG CAC AGA GC and the reverse primer sequence CTT CAG GGG CAA CAC GAA GC were used. β-actin forward primer CGG CTA CAG CTT CAC CAC CA and reverse primer CGG GCA GCT CGT AGC TCT TC were used as housekeeping genes.

### Quantification of gene copy number

The copies of delivered plasmid were determined using real time PCR. DNA was isolated from corneal fibroblasts using a DNeasy kit (Qiagen). The standard plot was prepared using a 10 fold serial dilution of 10^11^-10^6^ copies of sTGFβRII pCDNA3.1 plasmid. Forward primer ACG GTG CAG TCA AGT TTC CAC AAC and reverse primer ACA CAG ACT TCC TGT GGC TTC TCA were used for running the real time PCR with the following settings: 95 °C for 10 min, 40 cycles at 95 °C for 15 s, and 60 °C for 60 s.

### Quantification of protein expression due to delivered gene

Sandwich ELISA for sTGFβRII was done on the culture medium collected from sTGFβRII-transfected HCF. The collected medium was concentrated using a centrifugal Vacuum Concentrator (Labconco), and ELISA was performed following manufacturer’s instructions using a commercially available kit (R&D Systems, Minneapolis, MN).

### Cytotoxicity of polyethylenimine-DNA nanoparticles

The PEI-DNA nanoparticles cytotoxicity to HCF was evaluated measuring cellular proliferation with MTT, total cell counts with DAPI-staining and apoptotic cell death with TUNEL assays following vendors’ instructions. Briefly, for MTT assay (Promega), 5000 HCF were seeded in a 96 well plate and exposed to PEI-DNA nanoparticles mixture for 30 min, and at 2, 6, 12, and 24 h post-transfection cellular proliferation was quantified by measuring change in color intensity using spectrophotometer. To compare total cell counts in ±nanoparticles treated HCF, DAPI-stained nuclei at 200X magnification in six randomly selected, non-overlapping areas were counted. To determine cell death, cultures were fixed in paraformaldehyde, washed with PBS, subjected to TUNEL assay (Millipore), and TUNEL+cells in ±nanoparticles treated cultures at 200X magnification in six randomly selected, non-overlapping areas were counted.

### Statistical analyses

Results were expressed as a mean±standard error. Statistical analysis was performed using One way ANOVA and Tuckey’s test. The standard curve for the gene copy number and ELISA was subjected to regression analysis, and slope, intercept and correlation coefficient were calculated. The value of p<0.05 was considered significant.

## Results

### Formation of polyethylenimine-plasmid polyplex

**Figure 1:**
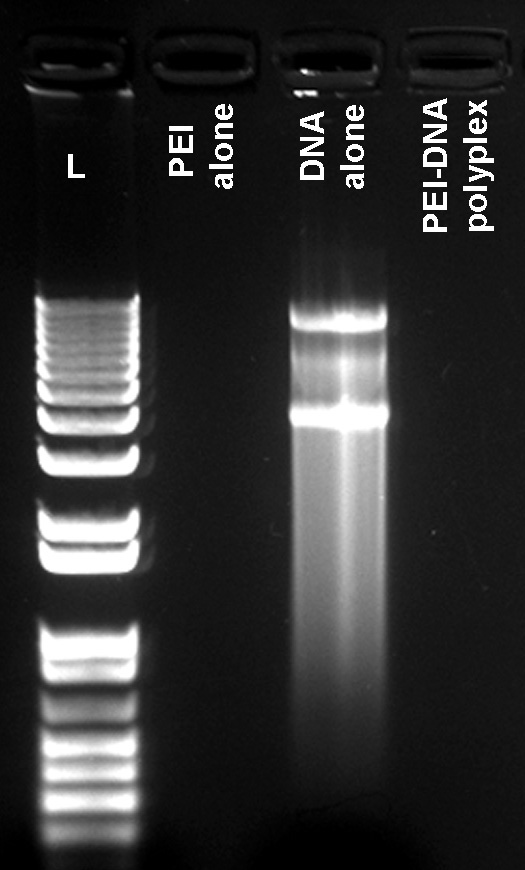
Agarose gel electrophoresis showing DNA migration and polyplex formation between PEI and plasmid. Plasmid alone showed DNA migration (lane-2) but no DNA migration was detected in polyplex (lane-3) or PEI alone (lane-1) on the gel. **L**: denotes 1 kb plus DNA ladder loading.

The formation of PEI-DNA polyplex was confirmed by inhibition of DNA migration using agarose gel electrophoresis. As detected in Figure 1, the PEI completely inhibited electrophoretic migration of DNA suggesting complex formation between the positively charged PEI and the negatively charged plasmid. PEI complexation to plasmid prevents the ethidium bromide dye from binding to the DNA resulting in no staining of the DNA band in the wells. The control, plasmid DNA alone, showed normal electrophoretic migration in gel and ethidium bromide staining (Figure 1).

### Determination of delivered soluble transforming growth factor–β type II receptor gene copies

**Figure 2:**
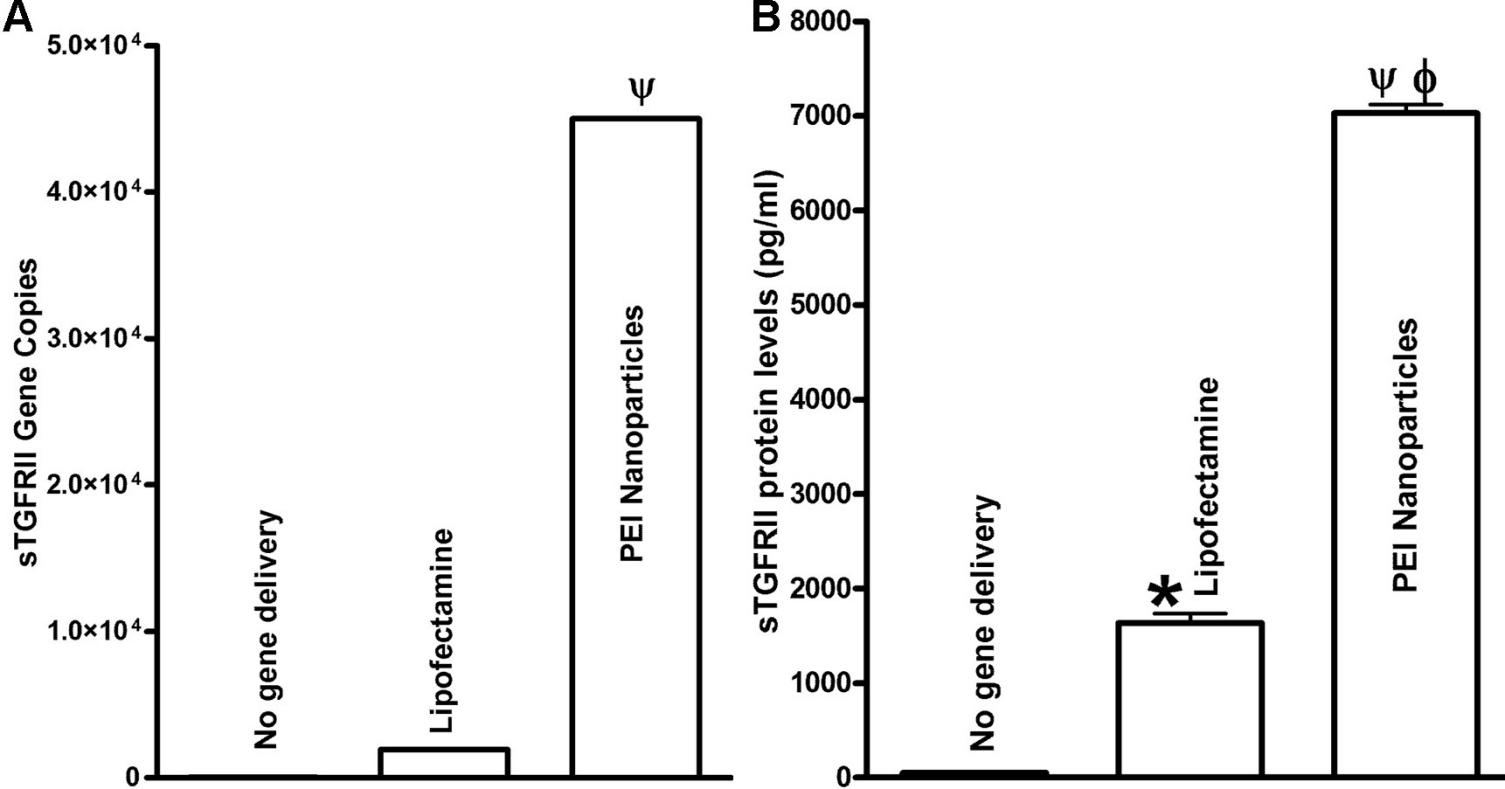
Real-time PCR. **A**: and ELISA **B**: showing quantification of delivered transgene gene copy number and protein levels in human corneal fibroblasts. The nanoparticle-transfected cultures showed high 4.5×10^4^ plasmid copies per µg DNA. This was statistically more than the lipofectamine or un-transfected cultures (ψ; p<0.001). Also, nanoparticle transfected cultures showed significantly high 7,030±87 pg/ml of sTGFβRII levels in ELISA assay of culture medium compared to un-transfected (ψ; p<0.001) and lipofectamine-transfected (ϕ; p<0.01) cultures. Lipofectamine-transfected HCF showed 1640±100 pg/ml of sTGFβRII (*, p<0.001 compared to un-transfected). The un-transfected HCF showed undetectable protein levels of sTGFβRII.

The sTGFβRII gene copies delivered into HCF with nanoparticles or lipofectamine were quantified with real-time PCR and are shown in Figure 2. Detection of 4.5×10^4^ sTGFβRII gene copies per µg DNA delivered with nanoparticles and 0.2×10^4^ sTGFβRII gene copies per µg DNA delivered with lipofectamine into HCF demonstrated high transfection efficiency of PEI-DNA nanoparticles (Figure 2A).

### Quantification of soluble transforming growth factor–β type II receptor protein

To ensure that nanoparticle-mediated sTGFβRII-Fc plasmid delivery results in successful gene transcription and secretion of sTGFβRII protein, the protein levels of secreted sTGFβRII in the culture medium of transfected HCF were quantified using ELISA assay (Figure 2B). The nanoparticle-transfected HCF showed a significantly high 7,030±87 pg/ml protein levels of sTGFβRII in the culture medium obtained from HCF transfected with PEI-sTGFβRII-Fc plasmid (p<0.001 compared to untransfected; p<0.01 compared to lipofectamine-transfected). Contrary to this, lipofectamine-transfected HCF showed 1,640±100 pg/ml of sTGFβRII in the culture medium (p<0.001 compared to untransfected). The naked vector transfected controls showed no detectable levels of sTGFβRII protein in ELISA. These results confirm successful transgene expression in the HCF transfected with PEI-DNA nanoparticles and suggest that tested nanoparticles are a more efficacious vector for HCF than the lipofectamine.

### Effect of soluble transforming growth factor–β type II receptor gene delivery on myofibroblasts and fibrosis

**Figure 3:**
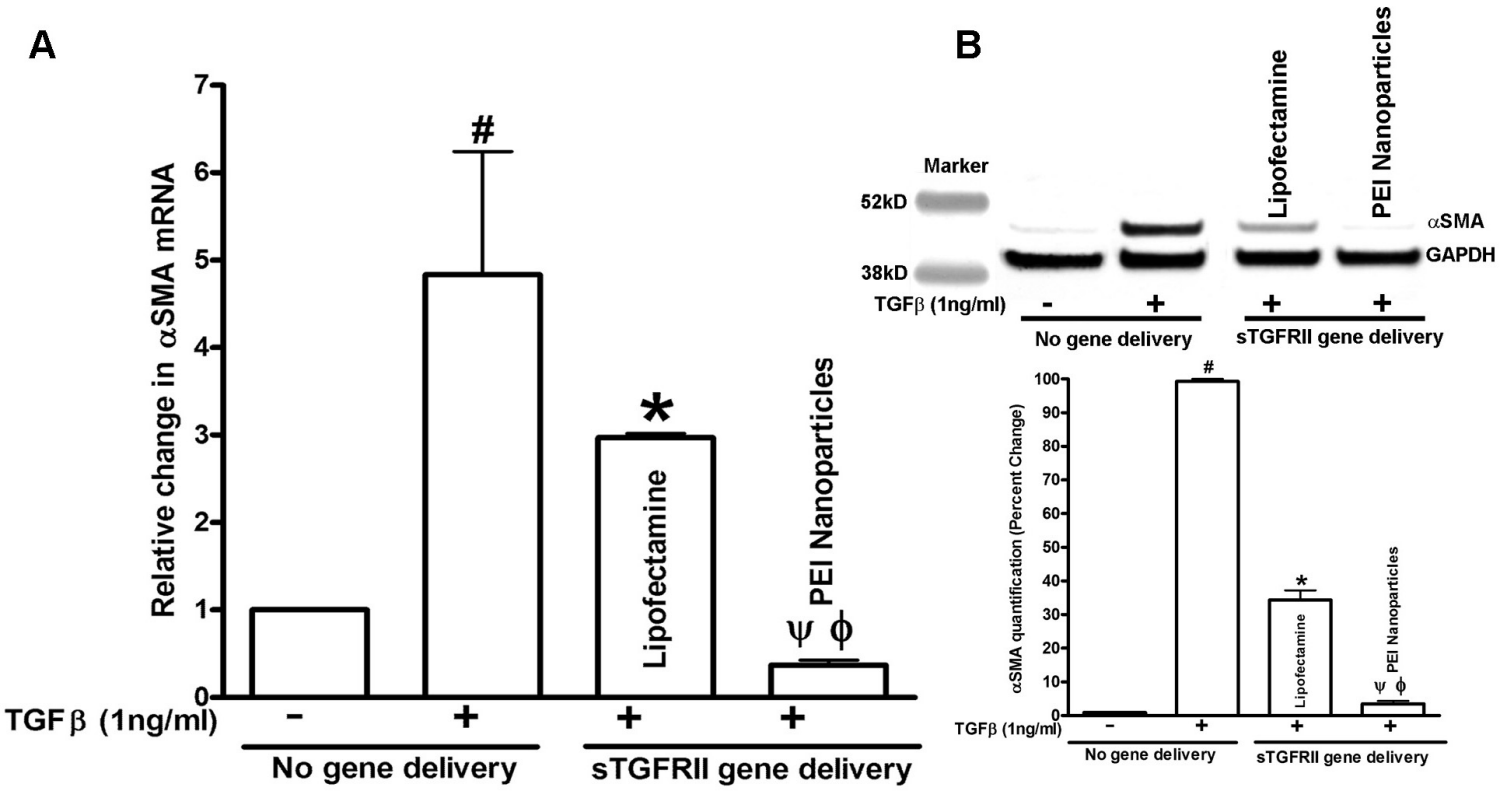
Quantification of αSMA mRNA. **A**: and protein **B**: levels in ±TGFβ1 treated HCF cultures showing anti-fibrotic response of sTGFβRII-Fc gene transfer. **A**: Real-time PCR showed that TGFβ1 treatment caused 5±1.4 fold increase in αSMA mRNA (#; p<0.01 than TGFβ1 untreated) and nanoparticle-mediated sTGFβRII-Fc transfection significantly lowered αSMA mRNA (0.36±0.08 fold, ψ; p<0.01 compared to TGFβ1-treated and ϕ; p<0.05 compared to lipofectamine-transfected). **B**: In western blotting an expected significant increase in αSMA levels were detected in TGFβ1-treated HCF over the TGFβ1-untreated HCF (#; p<0.001). Nanoparticles-mediated sTGFβRII-Fc gene transfer into HCF demonstrated a significant 96±3% decrease in αSMA levels (ψ; p<0.001 compared to TGFβ1-treated and ϕ; p<0.01 compared to lipofectamine-transfected). Lipofectamine-delivered sTGFβRII-Fc HCF used for comparison showed less than 60% decrease in αSMA expression.

**Figure 4:**
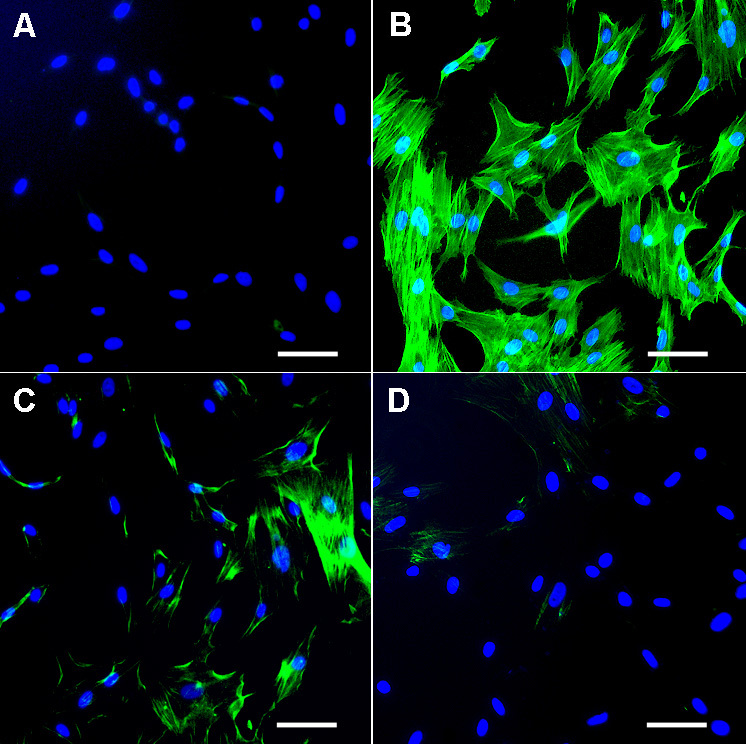
Immunocytochemistry showing the effects of nanoparticles-mediated sTGFβRII gene transfer on αSMA and myofibroblast inhibition. No αSMA+ cells were detected in un-transfected HCF grown in the absence of TGFβ1 **A**: and >80% αSMA+ cells were detected in cultures grown in presence of TGFβ1 **B**: The nanoparticles mediated sTGFβRII-Fc gene delivery into HCF decreased αSMA+ cells **D**: >90% compared to TGFβ1-treated (p<0.001) and >68% than lipofectamine-delivered sTGFβRII **C**: p<0.01. Scale bar denotes 50 μm.

The transformation of corneal fibroblasts to myofibroblasts has been identified as the primary event in corneal fibrosis development. Myofibroblasts are contractile, metabolically active and opaque cells containing intracellular microfilament bundles of F-actin and αSMA. To test the antifibrotic effect of nanoparticles or lipofectamine delivered sTGFβRII-Fc, the mRNA and protein levels of αSMA were quantified using real-time PCR (Figure 3A), immunoblotting (Figure 3B) and immunostaining (Figure 4). As seen in Figure 3A, TGFβ1 treatment to corneal fibroblasts caused a 5.0±1.4 (p<0.01) fold increase in the mRNA expression of αSMA, and a significant decrease in αSMA mRNA levels were detected in cultures that were transfected with sTGFβRII-Fc with nanoparticles (0.36±0.08 fold; p<0.01) or lipofectamine (3.3±0.5 fold; p<0.05) compared to TGFβ1 alone treatment. Indeed, the inhibitory effects were more pronounced in cultures in which sTGFβRII was introduced via nanoparticles instead of lipofectamine (Figure 3A).

Figure 3B shows the results of αSMA immunoblotting performed with protein lysates prepared from HCF cultures transfected with nanoparticle or lipofectamine and grown in ±of TGFβ1. Samples with no-TGFβ1 treatment showed a faint αSMA band, TGFβ1 treatment induced myofibroblast production resulting in a prominent αSMA band, whereas sTGFβRII-Fc-transfection arrested myofibroblast formation as indicated by the detection of a weak αSMA bands in nanoparticle or lipofectamine transfected samples (Figure 3B). The digital quantification of three independent western blotting experiments (Figure 3C) found that PEI-mediated sTGFβRII-Fc transfection caused a statistically significant decrease in αSMA (96±3%; p<0.001 compared to TGFβ1-treated), which was 30% (p<0.01) more than the lipofectamine transfection. Lipofectamine-mediated sTGFβRII-Fc transfection to HCF showed 68.0±1.4% αSMA reduction (p<0.001 compared to TGFβ1-treated). This data suggests that sTGFβRII gene delivery reduces corneal fibrosis and that the nanoparticle-mediated gene transfer was more efficient compared to lipofectamine’s.

The anti-fibrotic effects of sTGFβRII-Fc gene transfer were further investigated by αSMA immunocytochemistry (Figure 4). As expected HCF cultured in the absence of TGFβ1 under serum-free conditions showed minimal αSMA expression (Figure 4A) whereas TGFβ1 (1 ng/ml) treatment caused a robust myofibroblast formation as indicated by an intense αSMA immunostaining in cultures (Figure 4B). The lipofectamine-mediated sTGFβRII-Fc transfection showed 63±4% (Figure 4C) and nanoparticle-mediated sTGFβRII gene transfer inhibited 94±5% myofibroblast formation (Figure 4D). The quantification of αSMA carried-out by counting αSMA+ and DAPI-stained nuclei in 10 randomly selected 200X magnification fields found that sTGFβRII gene transfer accomplished with nanoparticles was significantly more efficacious than the TGFβ1 treated un-transfected (Figure 4B; 94±5%, p<0.001) or lipofectamine-transfected cells (Figure 4C; 43±4%; p<0.01).

### Effect of nanoparticles on cellular proliferation and cell death

**Figure 5:**
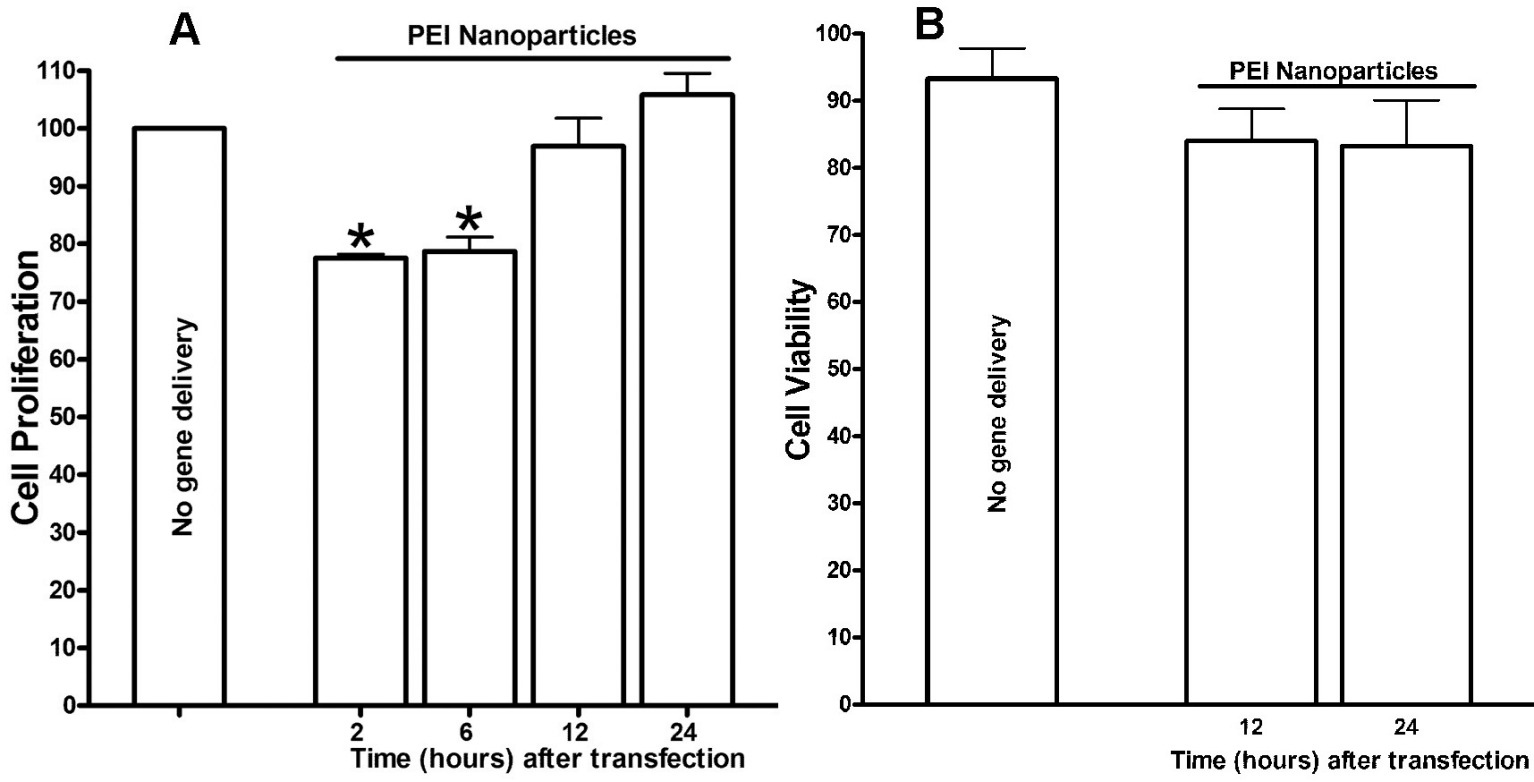
Quantification of MTT assay. **A**: and DAPI-stained nuclei **B**: data showing the effects of nanoparticles on cellular proliferation and survival/loss. **A**: Cell proliferation was quantified by measuring reduction of tetrazolium to formazan by live cells in MTT assay. The nanoparticles treatment to HCF did not cause overall impact HCF proliferation except showing early transient decrease in cellular proliferation at 2- and 6-h time points (22–24±3%; p<0.05) compared to untreated control. **B**: The DAPI-stained nuclear counting showed that nanoparticles are non-cytotoxic to HCF at 12 and 24 h.

Figure 5 shows the effects of nanoparticles (PEI-sTGFβRII-Fc plasmid) on cellular proliferation measured with MTT assay (Figure 5A) and total cell loss determined with DAPI-staining (Figure 5B) at various time points. As seen in Figure 5A, tested nanoparticles showed 22–24±3% (p<0.05) decrease in cellular proliferation up to 6 h. Nonetheless, this decrease in cellular proliferation was transient as no differences in cellular proliferation were detected between the nanoparticle-transfected and un-transfected HCF at 24 h post-treatment (Figure 5A). An expected 7%–9% loss of cells was detected in nanoparticle-transfected cultures compared to un-transfected cultures at 12- and 24 h post-treatment (Figure 5B).

**Figure 6:**
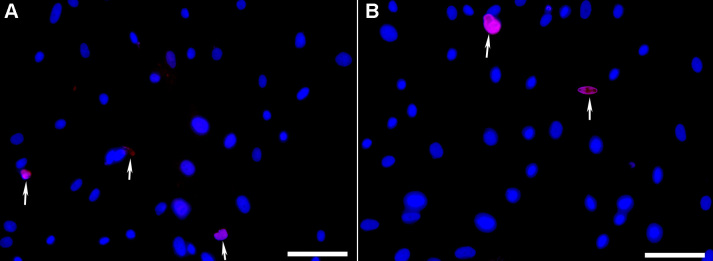
TUNEL assay performed 24 h after the PEI-DNA nanoparticles treatment showed that tested nanoparticles do not cause HCF death. No significant differences in TUNEL+ cells (Red) were seen in the untreated **A**: and nanoparticle-untreated **B**: HCF at 24 h. Nuclei are stained blue with DAPI. Scale bar denotes 100 μm.

Figure 6 shows results of TUNEL assay that primarily detects apoptosis and necrosis to a lesser extent. Detection of 2–5 TUNEL+ cells in 200X magnification field in nanoparticle-transfected (Figure 6A) and un-transfected (Figure 6B) cultures 24 h post-treatment suggests that PEI-sTGFβRII nanoparticles do not induce cell death.

## Discussion

Poor transfection efficiency of non-viral vectors for corneal cells is a limiting factor in the development of non-viral gene therapy for corneal diseases. Nanoparticles can serve as an efficient gene therapy vectors [1-6]. In this study, we report that PEI-DNA nanoparticles are efficient vector for delivering genes into corneal cells without compromising cellular proliferation, phenotype or viability, and that nanoparticle-assisted delivery of genetic material encoding for therapeutic gene, sTGFβRII-Fc chimeric protein secreted as a dimer, remarkably attenuates myofibroblast formation using an established in vitro corneal fibrosis model. Further, results of our study suggest that the tested nanoparticles offer greater gene transfer and therapeutic response compared to the commercial reagent, lipofectamine, in vitro.

A primary event in corneal fibrosis development is the transdifferentiation of quiescent corneal keratocytes to fibroblasts and myofibroblasts accompanied by increased deposition of collagen and matrix proteins [18,19]. After wound repair, myofibroblasts presumably disappear through apoptosis although the actual molecular mechanism of their disappearance is still unclear. Myofibroblast formation and haze development in cornea is largely mediated by hyper-TGFβ activity following corneal insult [20,21]. Additionally, TGFβ also stimulates the de novo synthesis of the extracellular matrix proteins, and thus further contributes to the corneal fibrosis and opacity development [23,24]. It is our working hypothesis that blocking of TGFβ or its signaling is a promising strategy to attenuate myofibroblast and scar formation in the cornea. We and others researchers have previously reported promising results to reduce corneal fibrosis by inhibiting TGFβ with antisense oligonucleotides, antibodies or genes like decorin, Smad7 etc [25-29]. However, a novel strategy to block TGFβ signaling in a TGFβ-specific manner is needed. Thus, we generated a vector expressing an ectodomain of the soluble type II TGFβ receptor fused to the Fc portion of human IgG and tested its potential for gene therapy using an in vitro corneal fibrosis model. The sTGFβRII blocks TGFβ signaling most probably by adsorbing TGFβ, although there is a possibility that it may also work as a dominant-negative receptor. Indeed, detection of high sTGFβRII protein in culture medium and a remarkable decrease in myofibroblasts not only supported our hypothesis but also demonstrated the promise of this approach for treating corneal scarring. We further postulate that high myofibroblast inhibition by sTGFβRII-Fc is due to the therapeutic chimeric protein produced as a dimer that binds and neutralizes TGFβ several fold more than its monomer form [32]. Preclinical animal studies have shown significant resolution of fibrosis in lung and kidney after sTGFβRII-Fc gene therapy delivered via adenovirus or non-viral vector [32-34]. Contrary to these findings, few studies did not find sTGFβRII-Fc gene transfer effective [35]. These conflicting reports suggest that anti-fibrotic effects of sTGFβRII-Fc are tissue-specific. We speculate that nanoparticle-mediated sTGFβRII-Fc gene therapy in the cornea in vivo will effectively reduce corneal opacity and effects will be long lasting due to the prolonged half-life of therapeutic chimeric sTGFβRII-Fc protein, which has shown extended in vivo half-life in various tissues [32-34]. However, its half-life in the cornea is yet to be determined.

Nanoparticles used in this study were prepared from PEI, which may cause cellular toxicity depending on their shape, size and molecular weight as branched PEI is reported cytotoxic to a variety of cells [8,9]. Conversely, linear PEI was found less cytotoxic and showed little damage to cell membrane and mitochondrial function or change in cellular viability and proliferation in many cell types [11,12,15]. The linear PEI-DNA nanoparticles, tested in present study did not alter human corneal fibroblasts proliferation, viability or induced cellular death. This suggests that PEI-DNA nanoparticles are not cytotoxic to corneal cells and warrants their in vivo testing. Another interesting observation is a proliferation slowdown of human corneal fibroblasts at an early 12 h period with these nanoparticles. Although, the relevance of this observation at this time is unclear, we postulate that this would have role in reducing myofibroblasts development in the cornea. It is well documented that active proliferation of quiescent keratocytes immediately after ocular injury plays an important role in corneal wound healing and leads to myofibroblasts and haze formation in the cornea [36,37]. It remains to be studied whether transient early arrest of corneal fibroblast proliferation by selected PEI nanoparticles will have any affect on corneal wound healing. Future in vivo studies will address this issue. In summary, this study demonstrates that PEI-DNA nanoparticles are potent and safe vector for introducing therapeutic genes into corneal cells to treat disorders, and sTGFβRII-Fc gene therapy delivered with these nanoparticles could be an effective approach to control corneal healing and treat corneal scarring in vivo.
